# Nondestructive Testing of the Miter Gates Using Various Measurement Methods

**DOI:** 10.3390/s20061749

**Published:** 2020-03-21

**Authors:** Mikolaj Binczyk, Przemyslaw Kalitowski, Jakub Szulwic, Pawel Tysiac

**Affiliations:** Faculty of Civil and Environmental Engineering, Gdansk University of Technology, 80-233 Gdańsk, Poland; mikbincz@pg.edu.pl (M.B.); przkalit@pg.edu.pl (P.K.); szulwic@pg.edu.pl (J.S.)

**Keywords:** structural health monitoring, nondestructive testing, comprehensive measurement methods, laser scanning, modal analysis, numerical model validation

## Abstract

When any problems related to civil engineering structures appear, identifying the issue through the usage of only one measuring method is difficult. Therefore, comprehensive tests are required to identify the main source. The strains and displacement measurements, as well as modal identification, are widely used in the nondestructive testing of structures. However, measurements are usually carried out at several points and confirm or exclude only one of many potential causes of the problem. The main aim of this paper is to identify the causes of miter gates’ excessive vibration. The research includes displacement measurements using a tachometer and a laser scanner, acceleration measurements connected with modal analysis, and calculations with the finite element method (FEM) model. The numerical model underwent verification regarding test results. Particular attention was paid to evaluate the practical use of a laser scanner for diagnosing miter gates. Unlike classical methods, it measures many points. The analysis eliminated a number of potential causes of excessive vibration and highlighted the field of excessive deformation. The identified anomaly could be associated with bearings’ misalignment after closing the door. This construction part should be subjected to further research using classical methods. The laser scanning has been proven to be a method that can only generally present the deformation of the structure.

## 1. Introduction

The aim of this paper is to indicate the causes of the poor operation of the water gates at the river barrage on the Vistula river in Wloclawek (the city located in the center of Poland). The problem had been identified by lock operators during normal operations of the chamber filling and emptying. It was related to a “sudden movement” that caused vibrations and shocks of the lock lower gates. The vibrations were transformed to other structural elements and were also noticeable in the control room building. The administrator of the facility failed to identify the main cause of the excessive vibration. Long-time and intensive vibrations can cause structural damage to a river barrage. Control structures related to the damaged strategic facilities pose a serious hazard and can cause enormous human and material losses. Due to these concerns, the facility owner commissioned an engineering evaluation regarding the cause of the vibrations and a proposed method of repair. Given the high importance of facilities in the form of locks on waterways, the detection of possible failures and appropriate monitoring of the facilities has a significant impact on the economic situation in the region. Their inspection usually involves the use of visual methods using surveying techniques combined with numerical calculations which are presented in [1]. In our opinion, empirical knowledge of the world today is primarily associated with the use of available methods of multidisciplinary significance. Therefore, to conduct the inspections, we used the technically approved method of structural monitoring described in [2] and extended it with some measurements and analyses. Combinations of surveying and dynamic measurements, along with numerical analyses, provided verification of the structure’s mechanical performance while performing miter gate operations. The approach which we present below suggests measurements of a few points, while the formulation of results takes place in the elimination process of possible structural defects. This manuscript is based on standard technical measurements of miter gates supported with laser scanning technology which is less often used in structural monitoring as compared with surveying techniques (because of its accuracy, precision, etc.). On the basis of our results, we believe that the reader can directly determine the usefulness of laser scanning technology for testing miter gates, as well as validate the results of our tests, thus determining the usefulness of testing the technical aspects of similar structures. By presenting the following considerations using this technology, we show our original contribution. Our results demonstrate the pros and cons of their use in the case of monitoring a miter gate and provide the basis for continuing research in this area as a multidisciplinary area combining civil engineering, remote sensing, mechanics or signal processing.

For the purpose of structure static analysis, first, a three-dimensional (3D) numerical model was performed using the finite element method (FEM) SOFiSTiK environment. Numerical modeling with FEM is commonly used for different issues related to construction and the determination of limit states of the structure of materials. FEM is an all-purpose tool used for structure numerical analyses. The basic concepts of the method are presented in [3]. Details of their implementation in the SOFiSTiK software are described in [4]. Although FEM is a popular, universal, comprehensive method, if it is possible, the FEM model should be verified by experiments. Actually, the study by [5] presented an example regarding the use of numerical calculations and verification thereof with studies on structure. The researchers proposed a composite whose bending strength was specified using a specially designed machine, which was confirmed with numerical tests. The numerical and laboratory test results differed slightly. Similar conclusions were drawn in a study by [6], in which some methods were compared. Usually, variance results are mainly from the different properties of the materials used (Young’s modulus, mass density), the impossible simulation of ambient conditions present during the performance of the tests (temperature, wind load), as well as the boundary conditions (joints, boundary conditions to the ground) [7]. Thus, if possible, researchers perform verification and validation of the established model to ensure that the performance corresponds to the real structure [8,9,10,11]. On the above basis, we were aware of possible variances between the calculating model and in situ tests. Therefore, measurements were performed with accelerometers to determine the structure’s natural vibrations. These measurements specified the structure’s dynamic parameters, namely, frequency, eigenforms, as well as damping. Any change of these parameters indicated structural damage. This property is often the basis of engineering evaluations regarding damages and their origin. Studies in [12,13,14,15,16,17,18,19,20] have specified some methods used for the detection of structural damages based on dynamic parameters. In addition, other nondestructive methods are known, such as inter alia model-based calculations and optimization data-driven algorithms [21,22], or mechanical wave propagation records [23,24]. Some researchers have combined the results of tests performed with accelerometers with FEM analyses for verification of structures, including football stadium roofing [25], elevated walkways [26,27], or cable stayed bridges [28].

To determine displacements during in situ tests, some surveying measurements were performed. Surveying services relate to a standard procedure, determining any displacement caused by different forces. Our tests employed two methods, i.e., tachymeter measurements and 3D laser scanning. The assessment of structure displacements resulting from test load using monitoring is specified in the work of [29], where surveying services were performed during static analyses regarding a railway tied-arch bridge. The methods employed for analyses of the results are specified in [30]. The general application of surveying services during tests of civil engineering structures is specified in [31], whereas [32] characterized tests, very similar to the ones described in this paper, which employed surveying services. The work in [32] relates to some properly selected satellite receivers that indicate average displacements approx. 20 cm vertically and 6 cm horizontally. Other interesting tests are specified in [33], where surveying measurements were performed using the IMU (inertial measurement unit). Measurements were related to bridge deformation during normal operation. Nevertheless, most methods related to static analyses are performed in terms of structure deformation analyses, namely [34], radar interferometry employed for building deformation analyses. In the case of less accessible places, where traditional solutions prove to be inefficient, the approach specified in [35] can be employed, where researchers use appropriate software and measuring instrumentation, namely, a laser scanner. These devices are widely used for structure diagnostic examination, as specified in works [36,37,38,39,40].

It is worth mentioning that measurements directly related to engineering structures are of great importance in the world. Any development of measurement methodology in the essence of a multidisciplinary approach means that we are able to use the acquired information for the general good of society. For the analysis of the literature in this area, special attention should be paid to [41], in which the authors rightly noticed the advantage of nondestructive methods. They noted, however, that in the case of high-precision results, they are not suitable for estimating the level of vibrations generated. In this way, we became aware that the measurement methodology presented must consist of complementary measuring instruments. The solution to the measurement problem related to vibrations was solved in [42], but without methods for measuring the displacements themselves. We began to realize that our solution must be reliable, while not generating high costs for the measurement itself. The inspiration for achieving such a result was the study by [43], where, based on large datasets, the authors successfully analyzed them without reducing the volume unnecessarily. Thus, they achieved satisfactory results, similar to those obtained in [44], for the case of turbine shaft torque. Therefore, knowing the complexity of the task, we hope that, in the sciences of engineering construction, it is recognized similar to the use of measuring techniques in the case of detection of corrosion and defects [45,46,47,48].

We divide the structure of our work into an introduction regarding the determination of the purpose of our work and the proposal of a comprehensive measurement service to determine the optimal conclusions with a numerical calculation base. Next, we present the concept of our methodology, together with an appropriate description of the sensors and instruments. Subsequently, we present the results of the measurements and analysis. Lastly, the obtained results are discussed, and appropriate conclusions are formulated.

## 2. Materials and Methods

**Figure 1:**
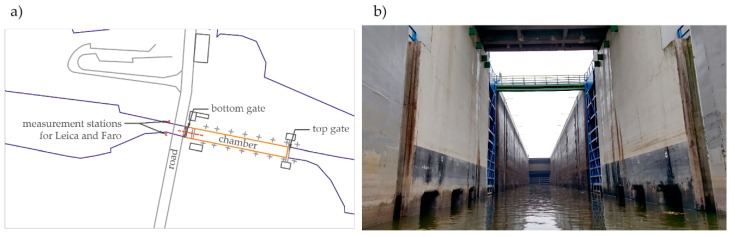
(**a**) Diagram of the lock in the Wloclawek river barrage; (**b**) View of the lock from the bottom side.

The lock was delivered mainly for navigation purposes, raising and lowering boats, ships, and other watercraft between different levels on the river. The maximum designed drop was 12.80 m, and the actual drop was 14.40 m. The lock, including roadstead, was located on the left bank. It was provided by a chamber with a size 115.00 × 12.00 m, at a downstream bar min. depth of 3.50 m (1.10 m at present). The lock featured a chamber, including the upper head and bottom head (Figure 1). The top closing of the lock was provided by a hydraulically driven segment that ensured water energy dissipation when filling the chamber with water. The bottom head was provided by a hydraulically-driven support gates. The upper and bottom head featured a monolithic reinforced-concrete structure. The chamber was provided with six dock sections, each 16.00 m long, provided with isolation joints. For repair purposes, steel flashboard beams were designed. If necessary, they were installed with a mobile crane. Chamber filling and emptying followed by-pass canals. Canal inlets and outlets were provided inside the heads at the two ends of the lock. By-pass canals carried the water towards the longitudinal chamber gallery, with openings located along the whole length, in the lower part of the chamber walls. By-pass canals were provided with flat gate valves.

Since lock erection, no works were performed that could have impacted on its structure or performance. In 2005, works related to renovation were delivered, including repair of concrete surfaces, sealing of expansion joints, delivery of head crown top surfaces and chamber walls, delivery of curbstones, and application of the concrete protective coating. During the following few years after the renovation, some irregularities were evident during gates’ operation, namely, sudden vibrations during water level change in the lock. The so-called sudden movement occurred only sometimes and was an accidental and occasional phenomenon.

**Figure 2:**
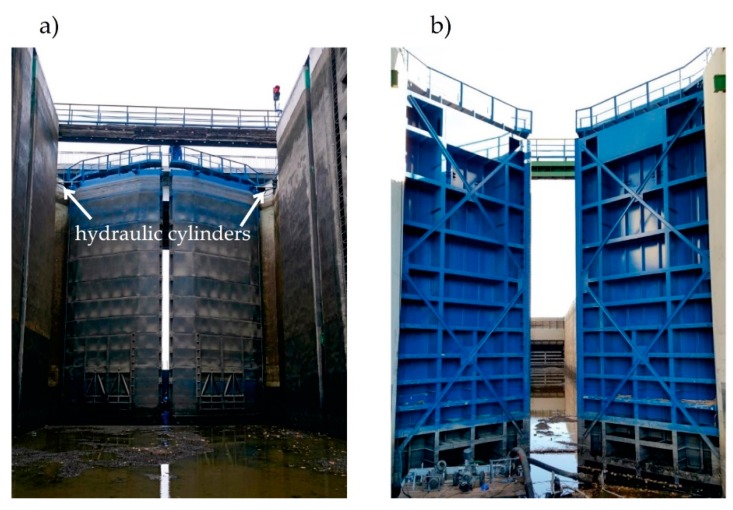
General view of the Wloclawek lock bottom miter gates. (**a**) From the side of the lock; (**b**) From the side of downstream.

The support gates of the bottom head were related to the movable double-leaf (Figure 2) feature with a frame structure, including I-section girders. The frame was covered with sheet cladding from the side of the lock. The gates’ elements were made from steel, with yield strength f_y_ = 235 MPa. Additional elements included bracing, rubber sealing, arched bearings, and rotating bearings, and top deck. Gates closing and opening were provided by hydraulic cylinders.

### 2.1. Observations Regarding Gates Operation

**Figure 3:**
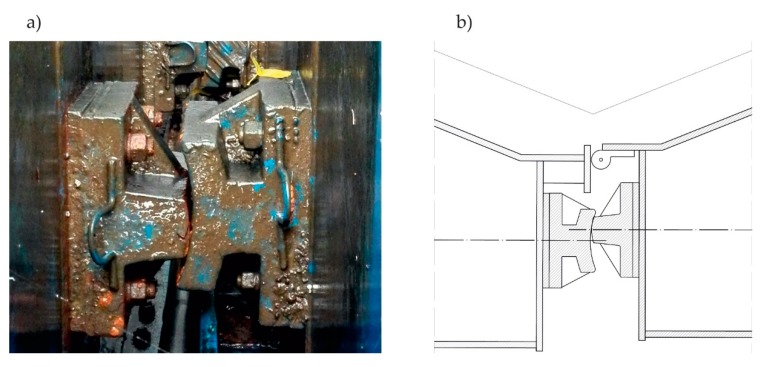
Observed misalignment of miter bearings. (**a**) Real view; (**b**) Scheme of the top view.

When totally emptied, the structure of the lock chamber underwent inspection based on visual examination, including photo records. The examination indicated misaligned miter bearings. Figure 3a shows a misaligned bearing that was observed. Figure 3b presents a scheme of this phenomenon.

**Figure 4:**
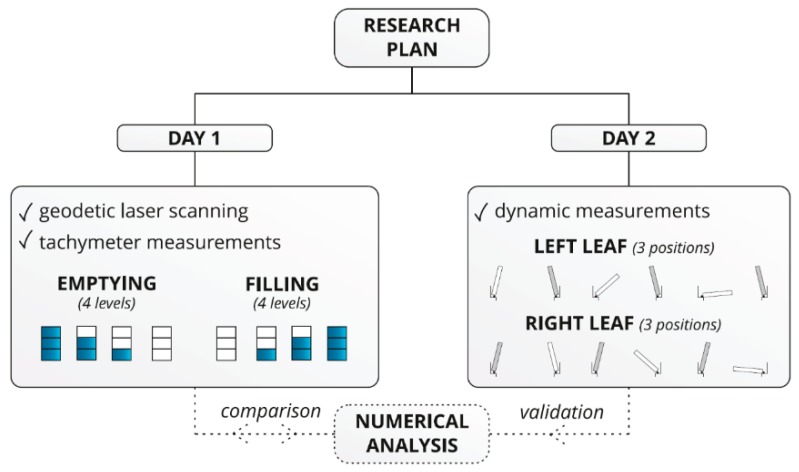
Diagram of tests regarding the lock in the Wloclawek river barrage.

Additionally, consultations followed with operators regarding any noticed cases related to interruption of lock filling and emptying. Operators reported some factors contributing to the sudden movement such as high temperature, strong wind blowing from the east (top water), and the irregular position of miter bearings after gates closing. According to the received information, the following goals of this research were set: To assess the static work of the lock during filling and emptying and assess work and condition of rotational bearings on the top and bottom. To achieve the goals, surveying and dynamical field tests were planned (Figure 4). Before the field tests, a numerical model was created. Static and modal analyses were executed. Static calculations were conducted for a comparison with surveying measurements. Dynamic calculations were conducted to evaluate the quality of the numerical model as comparing with modal model parameters with parameters identified in dynamic measurements. During the first day of the tests, the lock underwent emptying and filling, and each procedure included 4 steps. Following each step, the water level was recorded, including surveying measurements and laser scanning. During the second day of the tests, dynamic measurements were conducted. Six setups were done, 3 per each lock’s gates’ leaf. In each setup, the leaf had various opening angles, i.e., closed position, midposition between closed and opened, and fully opened position. 

### 2.2. Measurements and Methods 

**Figure 5:**
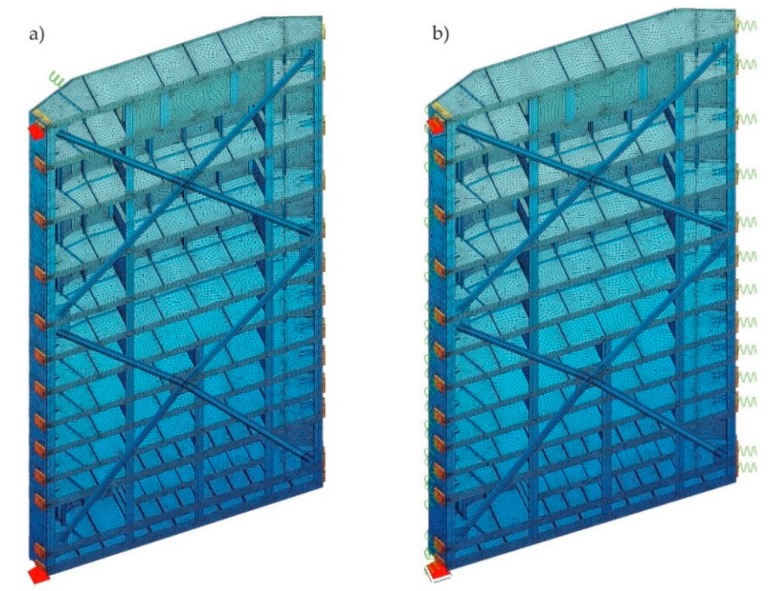
Visualization of the numerical models with different boundary conditions. (**a**) “Opened model”; (**b**) “Closed model”.

Firstly, static analysis was performed with the numerical model. For this purpose, the 3D model was created with FEM SOFiSTiK. Since the structural geometry of the gates’ leaves and the hydrostatic loading are symmetric about the leaf center line, only one of the gates’ leaves was modeled. Two-dimensional, four-nodal shell elements and one-dimensional beam elements were used for the construction [39]. The gates’ structural elements were elaborated pursuant to design documentation. However, the following assumptions and simplifications were applied in the model. Supports were taken into account by point constraints or elastic supports (springs). The effect of contact of the side and the bottom gaskets was not considered. Due to the different boundary conditions of the gates during opening (separated gates’ leaves) and after closing (connected gates’ leaves), two different models were established. The first one, named “opened model” (Figure 5a), provided simulation for open gates and allowed the identification of modal parameters. In this model, the rotational bearings were articulated supports. Because other bearings were not involved in the load transfer, they were omitted and only non-structural masses of those bearings and the upper deck were added to the model. The additional spring was mounted in place of the hydraulic cylinder. Its stiffness was established in the calibration process so that the natural frequencies of the modal analysis were consistent with those obtained from field tests. The second model, named “closed model” (Figure 5b), provided simulation for the closed gates and was purposed for static analyses. In this model, the rotational bearings were vertical, articulated, sliding supports. In place of the miter and the support bearings, rigid springs were mounted. Loadings to the “closed model” were provided with dead weight and the following water static pressure:

- A, maximum top water level (57.30 m above sea level) and bottom water zero level (41.80 m above sea level);
- B.1, top water level 1/3 and bottom water level + 1 m;
- B.2, top water level increase from 1/3 to 2/3 and bottom water level + 1 m;
- B.3, top water level increase from 2/3 to 3/3 and bottom water level + 1 m.

In both models, mesh convergence analysis was established. For the applied load with maximum magnitude, displacements in representative nodes were checked. The maximum dimension of the element was reduced iteratively until the displacements in subsequent steps did not change by more than 3%. In each subsequent step, the element size was reduced twice.

**Figure 6:**
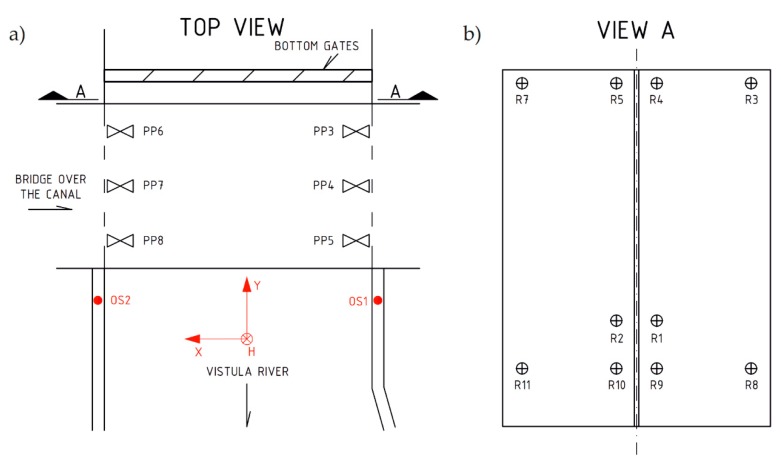
Sketch regarding a location of gates displacement measuring points. (**a**) Sketch regarding the location of control points and measurement positions, top view; (**b**) View from bottom water.

Surveying measurements were based on measurements performed with Leica TS30 (Leica Geosystems AG, Heerbrugg, Switzerland ) (with a precise mode about 0.6 mm + 1 ppm / typ. 7 s) at data recording precision of 0.1 mm. Figure 6 specifies the locations of control points (from PP3 to PP8), measurement positions (OS1 and OS2), and measuring points (from R1 to R10).

**Figure 7:**
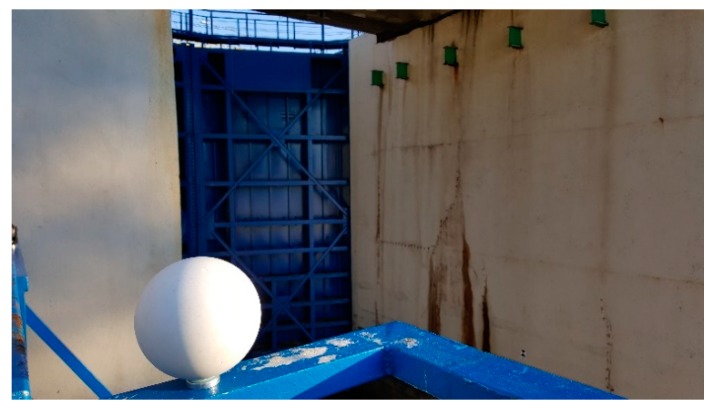
Representation of the surveying point during the tests.

For comprehensive survey and assessment regarding the entire gate deformation, measurements were performed with laser scanning. All measurements were performed using the Faro branded device, Focus 3D X130 (FARO Swiss Holding GmbH, Beringen, Switzerland) (with an accuracy of about 2 mm and a measurement speed up to 1 million points per second). Survey results allowed assessment regarding gates’ element deformation during lock filling. Before measurement, stabilization followed 6 flat marks (Figure 6a) to ensure proper coordination of the system, and 6 positioning balls (Figure 7) providing data records at the required accuracy. Due to the gates’ dimensions (12.50 × 16.00 m) and difficultly in accessing the gates, there was a need to take measurements from two different positions (from the left side “OS1” and the right side “OS2”, Figure 6) when performing the scanning.

**Figure 8:**
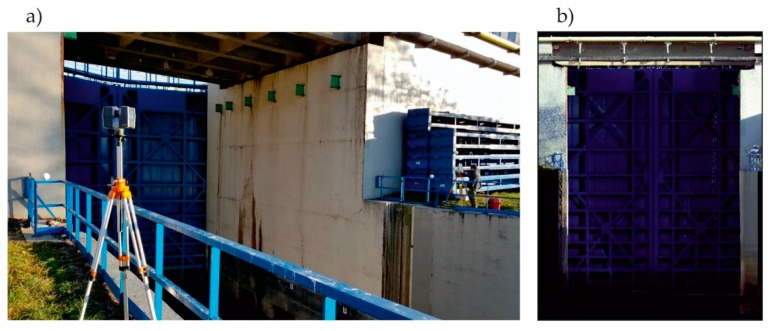
Laser scanner operation. (**a**) General view; (**b**) Scanning result, namely, a cloud of points, along with red-green-blue (RGB) color code.

Measurements with a tachymeter and a laser scanner were performed at the following 4 different water levels (Figure 4): (1) level 0/3, (2) level 1/3, (3) level 2/3, and (4) level 3/3. These were static measurements correlated with a constant water level in the lock. Each measuring series was taken for each of the two measuring positions, OS1 and OS2. Figure 8 includes the laser scanner operation and a cloud of points, along with the RGB (red-green-blue) color code.

**Figure 9:**
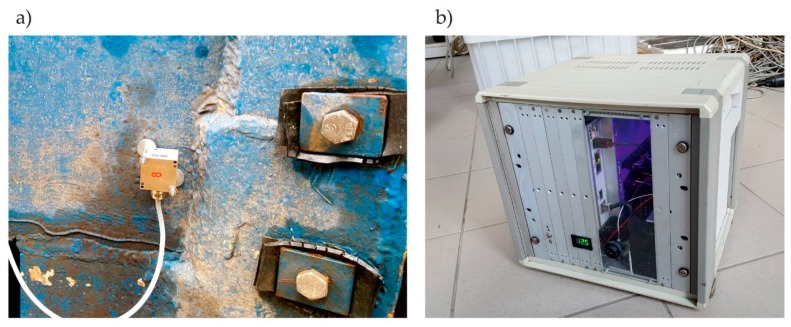
(**a**) Used tri-axis accelerometer TE 4332M3-002; (**b**) The individually improved HBM PMX Amplifier.

In addition, acceleration measurements were performed on the gates’ steel structure. As mentioned, the dynamic measurements had two main purposes, i.e., validation of the numerical FEM model and detecting of the unexpected movements of bearings. The measuring system consisted of the HBM PMX Amplifier (Hottinger Baldwin Messtechnik GmbH, Darmstadt, Germany) system, 4 MEMS (Micro Electro Mechanical System) Direct Current (DC) response accelerometers, and wires. The amplifier was connected by an Ethernet wire to a PC computer. HBM CatmanEasy (Hottinger Baldwin Messtechnik GmbH, Darmstadt, Germany) software installed on the PC was used in the configuration of the system and data acquisition. The used amplifier was designed for, and typically utilized in, industry control systems. However, after some modifications, its parameters allowed researchers to use it in laboratory and field tests. Improvements were carried out by the crew of the Field Test Laboratory of the Technical University of Gdansk. The amplifier was covered with a stiff box to protect it from mechanical damage and adverse weather conditions. Additionally, a battery was inserted into the box. Therefore, after connection with the PC computer (notebook type), it created a stand-alone, portable, 16-channels measuring system, which could be used for up to 8 hours without access to electricity. Another advantage was the elimination of redundant, additional vibrations coming from the electric generator’s work. It was especially significant to use low-noise sensors when low-magnitude vibrations are measured. Thus, the created acquisition system is very universal and convenient for field tests on bridges and other infrastructure constructions. In the system, following MEMS DC response, 4 to 20 mA signal output sensors were used as follows: 2 tri-axial accelerometers TE 4332M3-002 (TE Connectivity Ltd., Schaffhausen, Switzerland) and 2 one-axial accelerometers TE 4312M3-002. The following characteristic parameters of these accelerometers were: range ± 2 g, sensitivity 4.0 mA/g, frequency response 0 to 200 Hz, and natural frequency 700 Hz. The noise measured on the gates’ leaf, if no intentional load was applied, was in the range of ± 0.005 m/s^2^. The acquisition sampling rate was established to be 300 Hz. If predicted, interesting natural frequencies of the gates were below 30 Hz, it was evaluated as high enough to ensure the obtainment of all information about vibrations. Figure 9 shows the instrumentation for the acceleration measurements.

**Figure 10:**
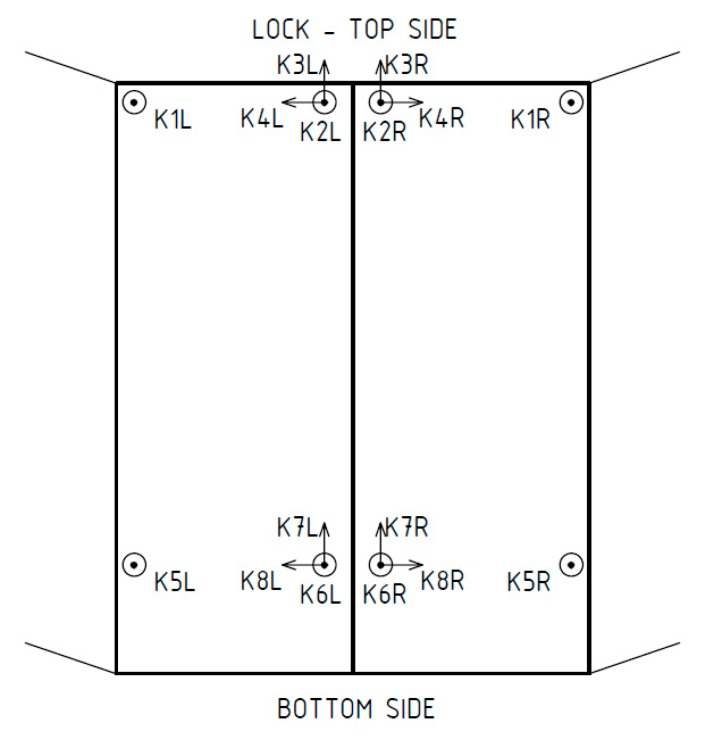
Location of the control points on the gates and the direction of the acceleration measurements.

In each of the 6 positions, the gates’ leaf was excited three times to natural vibrations, and then was left until vibrations damped. The measurements were carried out separately for the left leaf and separately for the right, as shown in Figure 4. Figure 10 specifies the location of the accelerometers placed on the gates’ steel structure. The same sensors were used for both leaves. Therefore, the letter "L" (left leaf) or "R" (right leaf) was added, as shown in Figure 10, for channel markings. The gates were excited to vibrations by shaking the bridge railing, by one of the researchers standing on the top of the gates’ leaf. The direction of the shakes was perpendicular to the gates’ surface. The starting frequency of shaking was approximately 4 Hz, and during excitation, it was adopted to the natural frequency. After obtaining acceleration amplitudes close to 1 m/s^2^, the shaking stopped. For analysis, when the shaking stopped and vibrations were damped to about 0.5 m/s^2^ was used as a signal.

During dynamic tests, time-domain free-response data were obtained. There exist numerous techniques for identifying the modal parameters from free vibration signals [49,50]. In this case, the ERA (eigensystem realization algorithm) was adopted [51,52,53,54]. It is an efficient method of modal parameter identification, confirmed in many real-world problems. A basic description of the method is presented below.

We consider the state-space representation of a discrete-time, linear time-invariant system in the form of:

(1) x(k+1)=Ax(k)+Bu(k)y(k)=Cx(k)+Du(k)

where x(k) is the vector of states, u(k) is the vector of system inputs, and y(k) is the vector of system outputs at the k-th step. A, B, C, D are the discrete-time state-space matrices. The order of the model is the number of components of the state vector x. For x(0)=0 and for the unit impulse excitation in all input elements:

(2) $$\{\begin{array}{cc} u\left(k\right)=1, & \mathrm{for}\;k=0\\ u\left(k\right)=0, & \mathrm{for}\;k>0 \end{array}$$

the results can be described as follows:

(3) $$Y_{1}=CB,\;Y_{2}=CAB,\;\ldots ,Y_{k}=CA^{k-1}B$$

These matrices in the sequence are known as the Markov parameters and describe the pulse response of the system. A system realization is the estimation of the set of matrices A, B, C from the Markov parameters for which the discrete-time model is satisfied. The minimum realization is the solution, where the order of the model is minimal.

In ERA, the first step is the formulation of the block Hankel matrix, composed from the Markov parameters in the form:

(4) $$H_{k-1}=\left[\begin{array}{cccc} Y_{k} & Y_{k+1} & \cdots & Y_{k+\beta -1}\\ Y_{k+1} & Y_{k+2} & \cdots & Y_{k+\beta }\\ \vdots & \vdots & \ddots & \vdots \\ Y_{k+\alpha -1} & Y_{k+\alpha } & \cdots & Y_{k+\alpha +\beta -1} \end{array}\right]$$

The shape of the Hankel matrix is equal (αm×β), where m is the number of output points (i.e., the number of sensors available for an identification).

The second step is the factorization of $H_{0}$ using singular value decomposition (SVD):

(5) $$H_{0}=R\Sigma S^{T}$$

where R and S are orthonormal matrices and Σ is the rectangular matrix (Equation (6)).

(6) $$\Sigma =\left[\begin{array}{cc} \Sigma_{n} & 0\\ 0 & 0 \end{array}\right]$$

where $\Sigma_{n}$ is a diagonal matrix with shape (n×n), and n is the model order. Due to the existence of noise in the acquired data, a minimal realization is obtained by eliminating relatively small singular values along the diagonal of $\Sigma_{n}$. Corresponding to eliminated values, rows and columns are removed, and from R and S arise $R_{n}$ and $S_{n}$, respectively.

System matrices, which are minimum realization, can be calculated as:

(7) $$\hat{A}=\Sigma_{n}^{-1/2}R_{n}^{T}H_{1}S_{n}\Sigma_{n}^{-1/2}$$

(8) $$\hat{C}=E_{m}^{T}R_{n}\Sigma_{n}^{-1/2}$$

where $E_{m}^{T}=\left[I_{m},\;0_{m},\;\cdots ,\;0_{m}\right]$, $I_{m}$ is an identity matrix of order m, and $0_{m}$ is a null matrix of order m. The accent $\left(\hat{\;\;\;}\right)$ means estimated values as opposed to true values. The modal parameters can be found by solving the eigenvalue problem:

(9) $$\hat{A}\Psi =\Psi \hat{\Lambda }\;$$

where Ψ is the matrix of eigenvectors and $\hat{\Lambda }$ is the diagonal matrix of corresponding eigenvalues. Eigenvalues of $\hat{A}$ are complex conjugates and each pair is associated with a mode of vibration. Before calculation of frequencies, a discrete-time system must be transformed to the corresponding continuous-time system using the relation:

(10) $$\Lambda_{c}=\frac{\ln\left(\Lambda \right)}{\Delta t}$$

Modal damping rates and damped natural frequencies can be obtained from the real and imaginary parts of the $\Lambda_{c}.$ In turn, the mode shapes can be obtained from the column vectors of the matrix $\hat{C}\Psi$. The ERA algorithm was implemented in Python. To assess the assumed model order, a stabilization diagram was created. To determine the diagram, the identification process was repeated with different (increasing) model order. If the modes are stable, they should remain constant in most iterations. Additionally, a filtered version of the stabilization diagram was generated [55]. It can be created using the criteria of evaluation of the solution modes. In our case, MAC (modal assurance criterion [56]) and MPC (modal phase collinearity [57]) were employed.

## 3. Results

**Figure 11:**
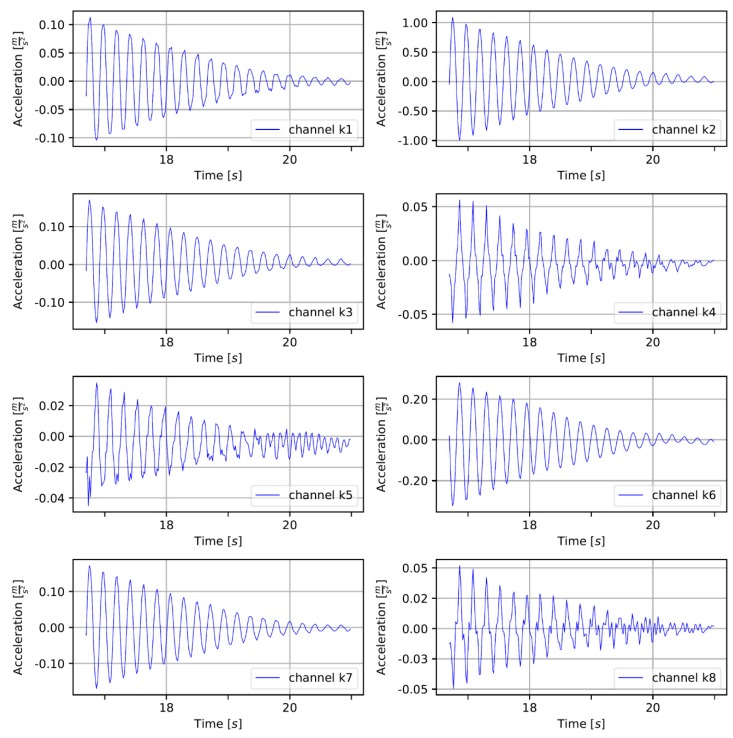
Acceleration measurements at points K1L to K8L during natural vibrations of the left gates’ leaf.

**Figure 12:**
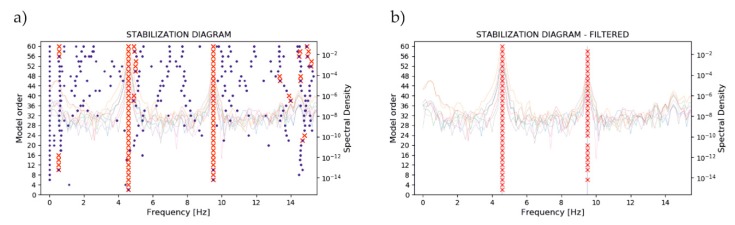
Stabilization diagram created for the gates’ left leaf. (**a**) Classic version; (**b**) Filtered version.

Before static analysis, the model underwent verification provided by comparing model eigenforms and eigenfrequencies with those identified in the field tests. Figure 11 shows an example of the measured accelerations in the gates’ left leaf in the middle position after shaking stopped. Each signal underwent basic, preparing processing, the constant component was removed, and the appropriate natural vibrations were cut from the whole series. To ensure better results in the ERA, in the range of lower frequency (0–15 Hz), the signal was downsampled to 60 Hz. To build the Hankel matrix, the following parameters of shape were used: α=12 and β=200. To identify stable modes, stabilization diagrams were used. Figure 12a presents an example of the classic version of the diagram, where all calculated poles are presented. On the chart, marked with red crosses, on the one hand, are poles which were evaluated as real as compared with results from previous iterations. On the other hand, the poles evaluated as numerical and artificial are marked with blue dots. Figure 12b shows a filtered version of the diagram. Here, only stable modes are shown. Additionally, on the background of the diagrams, a spectral density of signals is presented to demonstrate compatibility identified in the ERA modes and FFT results.

Among all measuring series, for both gates’ leaves, in the range to 10 Hz, two eigenmodes always occurred. Sometimes, in the results, one more stable mod appeared and its frequency was below 1 Hz. Subsequent analysis confirmed that it was the first mod of opened gates. However, it did not show up in all measurements, and cannot be taken into account for comparisons and diagnostics. Table 1 summarizes the identified natural frequencies for the second and the third mod for each leaf separately.

**Figure 13:**
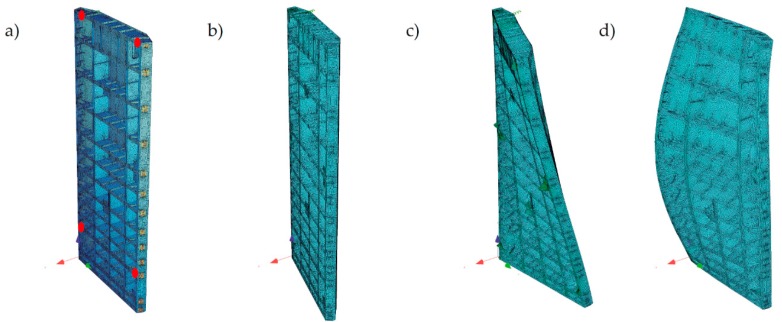
Frequency and eigenforms of the gates’ leaf according to the “opened model”. (**a**) Original geometry; (**b**) First eigenform, f = 0.51 Hz; (**c**) Second eigenform, f = 4.62 Hz; (**d**) Third eigenform, f = 9.46 Hz.

**Figure 14:**
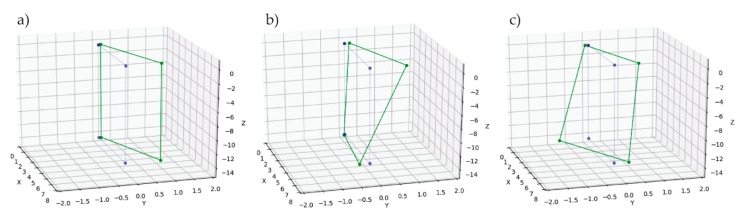
Identified in the eigensystem realization algorithm (ERA) eigenforms. (**a**) 1st; (**b**) 2nd; (**c**) 3rd. Blue points indicate the original positions of sensors. Green marked points displaced according to identified eigenform shape.

**Figure 15:**
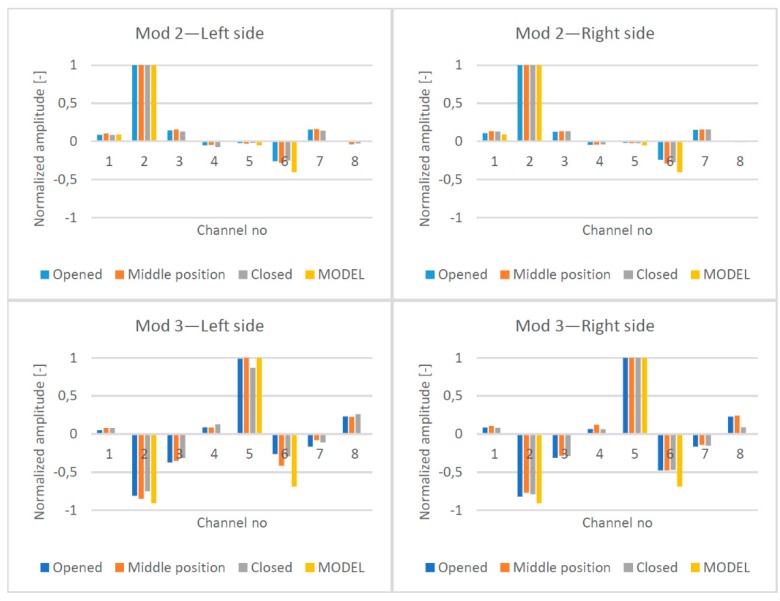
Comparison of the normalized eigenforms identified in three different positions of the gate’s leaves for the two identified mods. Yellow bars represent corresponding results from the numerical model.

The “opened model” was used for the purpose of modal analysis. The model was validated by adding non-structural masses to the top deck and the miter bearings. First, three eigenfrequencies and eigenforms were determined (Figure 13) and both, identified in the tests and determined in the FEM analysis, were similar. The ratio between that identified in the test frequency and that from the FEM model was in the range of 99–102% for the second mode and in range of 98–101% for the third mode. Those which were identified in the field test eigenforms for the direction perpendicular to the gates’ surface are presented in Figure 14. All eigenform coordinates were normalized. The linear connection between points was applied only for the visualization of mutual distances. For each leaf position and for each mod, the eigenforms were compared (Figure 15). All measuring points (K1 to K8) were included in the chart. Additionally, the normalized displacements of nodes were read from the numerical model for the second and the third mod. Appropriate nodes were chosen according to the location of measuring channels K1, K2, K5, and K6. The obtained frequencies and eigenforms for each mod were similar. Differences in frequencies and eigenforms, dependent on position, indicate that there were factors that slightly influence modal parameters. We discuss this phenomenon later. Although, from the aggregation of forms, it can be concluded that in all of the selected positions, the structure works in the same manner. Also, the displacements identified in channels related directly to potential bearings’ movement (K4 and K8) did not reveal any disturbing values. The above results indicate that the assumed stiffness and elements’ mass were established properly. The numerical model represents the real structure with a sufficient degree of precision. Therefore, there is the possibility of a comparison of calculated and measured at real structure displacements.

**Figure 16:**
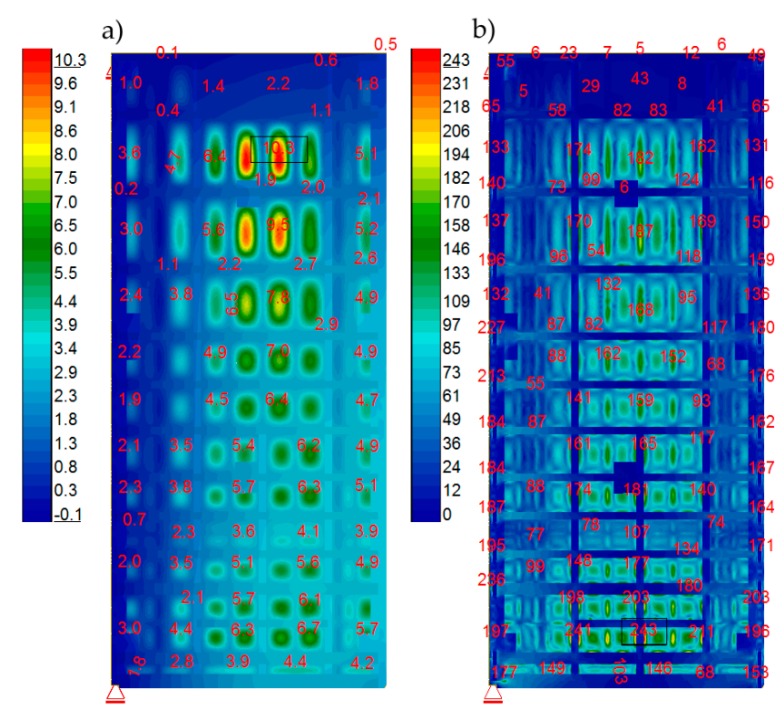
Calculation results regarding load A. (**a**) Displacements [mm] at water thrust direction; (**b**) Maximum von Mises stresses [MPa].

**Figure 17:**
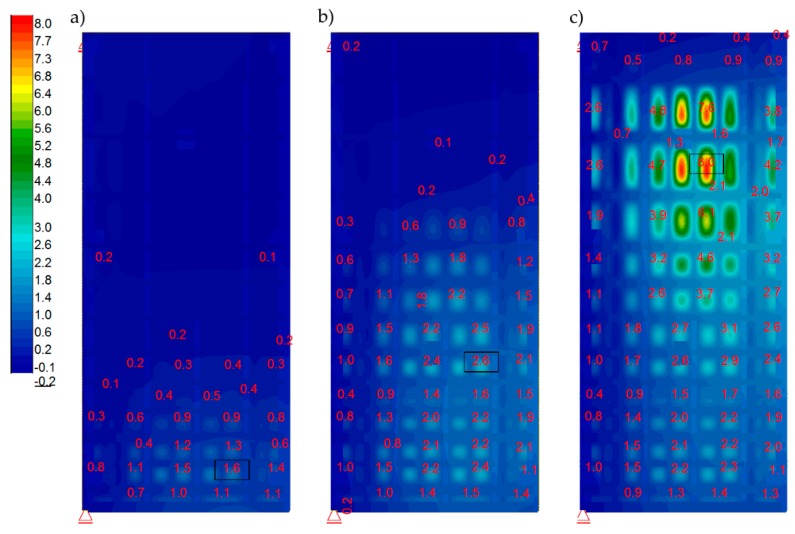
Calculated displacements [mm] at water thrust direction for respective loads. (**a**) B1; (**b**) B2; (**c**) B3.

Afterward, the verified FEM model was loaded with static water pressure at different water levels in the lock. A “closed model” was used for this analysis. The calculated load cases were as specified previously: A, B1, B2, and B3. Figure 16 specifies the results for Case A. This case represented a simulation regarding the lock gates under maximum load. It was employed to determine the maximum stress of structural elements. Calculated von Mises stresses for frame girders were not exceeded (Figure 16b). Values over 235 MPa were recorded for sheet cladding at joints with girders. They were local and resulted from the connection of the frame and the sheet in the numerical model. To obtain accurate results for these areas, a detailed numerical model was required. The load cases B1, B2, and B3 represented in-between situations that occurred during emptying and filling the lock. Actually, they corresponded to phases assumed for measurements with the tachymeter and laser scanning. Figure 17 shows the calculated displacements of the structure obtained during particular lock filling phases.

During chamber emptying, measurements with a tachymeter were performed at the following points: R1, R2, R3, R4, R5, and R6. During chamber filling, measurements with a tachymeter were performed at the following additional points: R7, R8, R9, and R10. Reading was performed four times (the following emptying, following filling, and two in-between measurements). Calculations regarding the displacement of measuring points at X, Y, and H direction for emptying were based on the difference between final and initial coordinates (the difference between water levels 3/3 and 0/3). Calculated displacements during filling were based on the difference between 1/3 and 3/3 water levels. The following tables include displacement measurements (Table 2 and Table 3).

**Figure 18:**
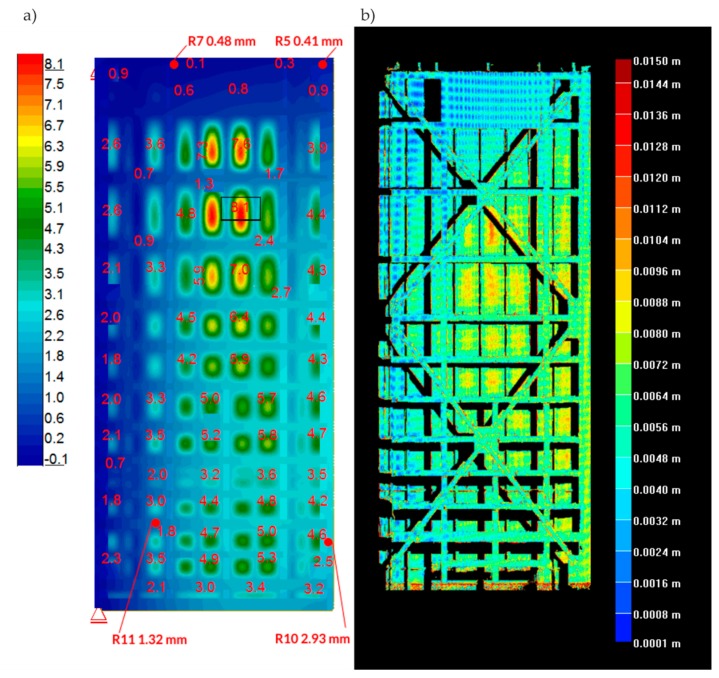
Comparative analysis regarding displacements at Y direction from. (**a**) Numerical model (mm); and (**b**) Laser scanning (m), top water increases from the 1/3 to the 3/3 level.

Note, at minimal water level, the minimal opening of the lock gates followed as a result of wind. During the initial filling phase, the displacement was also affected by the deformation of the gaskets. This fact explained the difference between dY displacements during filling and emptying. In addition, because of this, the diagram regarding top water increases from the 1/3 to the 3/3 level was used, as the most representative for the comparative analysis of displacements. The comparative analysis was performed regarding displacements at Y direction (horizontally at longitudinal direction) obtained during surveying measurements and laser scanning against numerical calculations. Figure 18 includes graphics of the results. On the map of displacements calculated with the numerical model (Figure 18a), four points are marked. The exact values of the calculated displacements are given. At these points, the measurement was performed with the use of a tachymeter. Table 4 shows a comparison of the calculated and measured displacements. Attention should be paid to two points (R4 and R5), for which the differences are in the order of several hundred percent. For the remaining points, the differences are much smaller. In Figure 18b, laser scanning results are presented. They were calculated by subtracting the coordinates of the two point clouds from two different measurements.

## 4. Discussion

Dynamic measurements in different positions of the gates’ leaves do not indicate any relevant displacements of the support bearings. A small difference between the identified, normalized modal displacements can be a consequence of adherence of the gasket to concrete elements. Differences occur, especially in the case of point K6, which is located near the bottom part of the gates, where the gasket is mounted. In addition to this, eigenfrequencies and eigenforms confirm matching between real structure and the numerical model.

During lock filling and emptying, the gates’ operation followed under stress and the numerical analysis did not indicate any excessive stress. 

The comparative analysis was performed regarding measurements and calculations of structure displacement resulted from water level change from 1/3 to 3/3. Horizontal displacements, at a direction parallel to the gates’ surface obtained based on the numerical model and the measurements with the tachymeter, were similar. Insignificant variance resulting from gates additional displacements resulted from pressure and gasket deformation at the initial phase during lock filling, which is a natural effect. Thanks to the miter gasket’s deformation the sealing of gates was provided. The numerical model did not include this phenomenon; only points close to the miter axis indicate an anomaly. Displacements measured with tachymeter in points R5 and R4 (miter top corner) are significantly different than those obtained with the numerical model. However, the remaining results indicate that structure operation was consistent with the numerical model (design input). Therefore, we could exclude any significant damage to the main structural elements. 

The displacements indicated by laser scanning were bigger than the results obtained using two other methods. The work provided an example regarding measurement accuracy difference during the elaboration of differential maps [58]. Additionally, water level changed slightly in the lock during laser scanning that featured a measurement period significantly longer (about seven min) than the one with the tachymeter.

**Figure 19:**
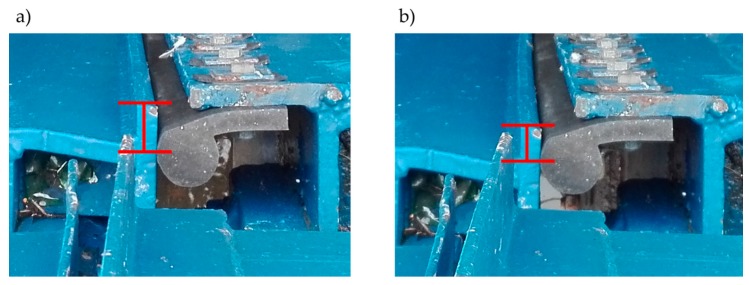
The shift between the gate’s leaves. (**a**) Position after sudden movement; (**b**) Position before sudden movement.

In summary, the visual examination and analysis regarding surveying measurements, vibration measurements, and laser scanning indicate that miter gates work properly, although all of the field tests were conducted without the occurrence of sudden movement. Thus, we concluded that only in the case of normal conditions does the operation of the gates work properly. All of the comprehensive measurements allow researchers to conclude that the rotating bearings work without redundant lose, and if sudden movement does not occur, the miter gates are characterized by elastic behavior. The differences between the measured and calculated displacements in the R5 and R4 points could indicate that boundary conditions close to these points can vary in structure and in the numerical model. During the visual examination, misaligned positions of the miter bearings were noticed. Unfortunately, no sudden movement was observed during the measurements. However, photographic documentation was taken before and after the example of sudden movement. Figure 19 shows the mutual position of the left and the right leaves after and before a sudden movement. The difference in the position of the leaves is marked with a red line. During a sudden movement of the miter gates, the miter bearings shifted. In [59] a similar problem was presented, which occurred in the case of the Red River Lock and Dam no 1. In [1], the authors also paid attention to the importance of adherence of the bearings for the appropriate distribution of loads. In our case, there is a possibility that the miter gates before filling are closed with misaligned miter bearings. It is hypothesized that, subsequently, the water level increases against the gates, and at some point, the pressure causes the leaves’ self-adjustment. The gates final closing follows suddenly and results in a significant horizontal force transfer onto other adjacent structural elements, consequently, increasing vibrations. 

## 5. Conclusions 

The research of the analyzed structure was performed using multiple methods that allowed assessment regarding different performances of the structure. The comprehensive analysis of all results enabled us to exclude some potential causes of excessive vibrations. Dynamic measurements were used to assess the condition of rotational bearings and confirmed their lack of redundant lose during operation. The correct operation of the rotational bearings was checked by comparing the eigenforms of the gates’ leaves in different opening positions. Additionally, thanks to the dynamic results, the numerical FEM model could be validated. The tachymeter measurements provided information about the generally elastic behavior of the gates during operation. Although, as compared with the FEM results, they also indicated points at which behavior is inappropriate. Deformations of these parts of the structure (near points R4 and R5) probably are caused by misalignment of miter bearings. The results from laser scanning are consistent with the FEM results in terms of direction and shape of deformation, also in both cases, an order of magnitude of displacements are similar. The laser scanning has been proven to be a method that can only generally present the deformation of the structure. Due to the subtraction of coordinates from two different point clouds, the accuracy of this measurement is insufficient to assess displacements in detail. In the authors' opinion, laser scanning can be used to measure displacements if they are significantly bigger than error arising from point clouds subtraction. However, it should be pointed out that all of the conducted measurements are significantly faster and cheaper in execution than is typical for cases utilizing strain gauges. Additionally, all of the employed methods provided mutual verification. The new combination of comprehensive measurement methods in nondestructive testing of the miter gates excluded some potential reasons of excessive vibrations. Nevertheless, further investigation should focus on researching how misalignment of miter bearings influences sudden movement existence. A possible option is to utilize inductive sensors between miter bearings to acquire mutual displacements. Another one is to use strain gauges and to detect indirectly the bearings without adherence.

## Figures and Tables

**Table 1:** Mean identified frequencies for each position.

Gates Leaf	Mod 2—Frequency (Hz)			Mod 3—Frequency (Hz)		
	Opened	Mid	Closed	Opened	Mid	Closed
**Left**	4.698	4.657	4.671	9.260	9.255	9.317
**Right**	4.706	4.573	4.594	9.301	9.498	9.548

**Table 2:** Emptying related displacements.

NR.	dX (mm)	dY (mm)	dH (mm)
R1	–0.6	5.2	1.1
R2	0.6	5.6	–0.1
R3	1.3	0.2	0.3
R4	–0.2	4.0	0.2
R5	1.2	5.0	0.3
R6	0.3	1.1	0.4

**Table 3:** Filling related displacements.

NR.	dX (mm)	dY (mm0	dH (mm)
R1	–1.5	–4.2	0.6
R2	–2.5	–4.4	0.5
R3	–2.9	–0.7	–0.3
R4	–2.3	–2.4	–0.1
R5	–2.1	–3.2	–0.4
R6	–1.4	–0.3	0.0
R7	–1.8	–2.8	–0.6
R8	–1.4	–4.0	0.6
R9	–2.5	–4.2	0.4
R10	–1.2	–0.8	0.2

**Table 4:** Comparison of the calculated and measured displacements.

NR.	dY_FEM_ (mm)	dY_tachymeter_ (mm)	dY_tachymeter_ / dY_FEM_ (%)
R3	−0.48	−0.7	146%
R4	−0.41	−2.4	585%
R7	−1.32	−2.8	212%
R8	−2.93	−4.0	137%
R5	−0.41	−3.2	780%
R6	−0.48	−0.3	63%
R9	−2.93	−4.2	143%
R10	−1.32	−0.8	61%
